# Posterior Fossa Hemorrhage Following the Use of Low-Molecular-Weight Heparin: Lessons Learned and Recommendations for the Treatment and Prophylaxis of Postoperative Venous Thromboembolism

**DOI:** 10.7759/cureus.15404

**Published:** 2021-06-02

**Authors:** Komal Naeem, Malika Bhargava, Michael Bohl, Randall W Porter

**Affiliations:** 1 Department of Neurosurgery, Barrow Neurological Institute, Phoenix, USA

**Keywords:** craniotomy, posterior fossa hemorrhage, posterior fossa lesion, prophylactic and therapeutic anticoagulation

## Abstract

Introduction

Venous thromboembolism (VTE) is the most common preventable cause of morbidity and mortality among neurosurgery patients. Several studies have concluded that the use of chemical prophylaxis among patients undergoing a craniotomy reduces the incidence of VTE, and it is presumed to be safe. However, these studies do not differentiate between a supratentorial and posterior fossa craniotomy. Furthermore, the prophylactic or therapeutic use of low-molecular-weight heparin (LMWH) has been reported to increase the risk of intracranial hemorrhage. In this study, we describe the clinical details and outcomes for all patients who underwent posterior fossa craniotomy and developed posterior fossa hemorrhage secondary to postoperative use of LMWH during the study period. We also propose recommendations pertaining to postoperative heparin use after posterior fossa surgeries.

Methods

Data were retrospectively collected for patients presenting with posterior fossa hemorrhage following anticoagulant use among those who previously underwent posterior fossa craniotomy by the senior author (R.W.P.) from January 1, 2011, through December 31, 2018.

Results

We identified five patients who experienced postoperative hemorrhage while receiving LMWH in the initial setting of posterior fossa craniotomy. After hemorrhaging, four patients had low Glasgow Outcome Scale (GOS) scores (≤3) and failed to return to their baseline neurological status. These four patients had a Glasgow Coma Scale (GCS) score of 15/15 in the immediate postoperative period and received heparin within 72 hours of surgery.

Conclusions

Based on our findings, there is a possible association between the increased risk of hemorrhage and the early postoperative use of LMWH. The debilitating outcomes among the majority of these patients warrant the cautious use and further investigation of postoperative LMWH to appropriately quantify the risk. Further comparative studies with a larger sample size are required to provide insight into the pathophysiology of our findings.

## Introduction

Venous thromboembolism (VTE), defined as the formation and migration of thrombus in the circulatory system, is said to be the single most significant preventable cause of morbidity and mortality among neurosurgical patients. VTE is classified as deep venous thrombosis (DVT), pulmonary embolism (PE), or both, and it occurs in the setting of Virchow’s triad: venous stasis, hypercoagulability, and endothelial injury [1-3]. The reported incidence rates of DVT and PE among neurosurgical patients are 0-34% and 0-3.8%, respectively. Brain tumors are correlated with the increased risk of VTE and, among patients with brain tumors, the incidence rates of DVT and PE have been reported to be 24-30% and 0-5%, respectively [2,4-10]. Chemoprophylaxis with low-molecular-weight heparin (LMWH) has been found to be the most efficacious method for VTE prevention when compared with unfractionated heparin (UFH) and mechanical prophylaxis (MP). However, this treatment comes with an increased risk of hemorrhage; the odds ratio for hemorrhage has been reported to be as high as 6.38 [95% confidence interval (CI): 0.58-69.91] when compared with placebo [4]. Postoperative hemorrhage following craniotomy, especially in the posterior fossa, can be catastrophic for patients [2,11]. Neurosurgical patients are at an increased risk of developing VTE, primarily because of increased venous stasis and hypercoagulability. Causes of this increased risk include underlying malignancy, increased operative times, postoperative or posttraumatic immobility, transient or permanent neurological deficits restricting patients’ mobility, and prolonged hospital stays [1-3].

The safety and efficacy of anticoagulation medication following cranial surgery have been studied widely, with several conflicting results reported. Some studies have concluded that it is safe to initiate LMWH in the early (from day one) postoperative period [5,12-15], whereas other studies have contradicted these findings [2,16-20]. The evidence-based clinical guidelines by the American College of Chest Physicians report the highest level of evidence for anticoagulation use as grade 2C (weak recommendations) [21]. Furthermore, existing evidence on the safety and efficacy of heparin products following surgery for brain lesions groups all lesions together and does not differentiate between supratentorial and posterior fossa lesions [5,12-20,22].

A retrospective study that evaluated the safety and efficacy of chemoprophylaxis following vestibular schwannoma (VS) resection found a higher rate of hemorrhage associated with the use of chemoprophylaxis when compared to mechanical compression devices (p<0.001). However, no significant reduction in the incidence of VTE was associated with chemoprophylaxis (p=0.32) [2]. The literature reports lower rates of VTE associated with posterior fossa surgery compared with other cranial surgeries, whereas both posterior fossa surgery and other cranial surgeries are associated with similar rates of intracerebral hemorrhage (ICH) [22,23]. These results highlight the importance of outlining VTE prophylaxis guidelines specific to posterior fossa surgery. The perilous consequences of both PE and hemorrhage following posterior fossa craniotomy render the decision regarding the use and timing of chemoprophylaxis highly challenging.

In this article, we share our experience and lessons learned with respect to chemoprophylaxis for VTE following posterior fossa surgery. We identified patients who experienced hemorrhage after undergoing posterior fossa tumor resection and received anticoagulation for VTE prophylaxis or treatment. Our aim is to examine the treatment outcomes and recovery time related to ICH secondary to prophylactic use of the anticoagulant LMWH. We also present our recommendations regarding the safe and effective use of anticoagulation for patients undergoing posterior fossa surgery.

## Materials and methods

A retrospective analysis was performed from January 1, 2011, to December 31, 2018, and data were collected for all patients who had posterior fossa surgery for tumor resection that were treated by the senior author (R.W.P.) and who developed symptomatic posterior fossa hemorrhage after receiving prophylactic or therapeutic anticoagulation. Patients with vascular lesions and trauma were excluded. We collected preoperative and postoperative details with emphasis on type, dosage, and timing of initiation of prophylactic and/or therapeutic anticoagulation, treatment outcomes, and post-hemorrhage recovery. All cases were followed up until the end of the study period. We also discuss the recommendations implemented by the senior author in 2015 for anticoagulation use following posterior fossa surgery. This study was approved by the St. Joseph's Hospital and Medical Center Institutional Review Board (IRB number: PHXB-15BN116).

## Results

We reviewed a series of 246 posterior fossa tumor resections performed by the senior author between January 1, 2011, and December 31, 2018, and identified five cases of posterior fossa hemorrhage following the prophylactic or therapeutic use of anticoagulants (Table 1).

**Table 1:** Demographic, preoperative, and postoperative details of five patients who developed intracranial hemorrhage after receiving therapeutic or prophylactic dose of low-molecular-weight heparin ^a^In addition to common risk factors for DVT: tumor and recent surgery; ^b^in addition to mechanical prophylaxis →: followed by, or subsequently; BMI: body mass index; CPA: cerebellopontine angle; DVT: deep venous thrombosis; EVD: external ventricular drain; GCS: Glasgow Coma Scale; GOS: Glasgow Outcome Scale; ICH: intracranial hemorrhage; IVC: inferior vena cava; IVH: intraventricular hemorrhage; L: left; M: male; MRI: magnetic resonance imaging; N/A: not applicable; PE: pulmonary embolism; POD: postoperative day; PY: pack-years; R: right; SAH: subarachnoid hemorrhage; SCD: sequential compression device; SDH: subdural hematoma; SNF: skilled nursing facility; UFH: unfractionated heparin; VPS: ventriculoperitoneal shunt; VS: vestibular schwannoma; VTE: venous thromboembolism

Patient	Age (Years), Sex	Risk Factors for VTE^a^	Diagnosis; Surgical Approach; operative time (min)	VTE Prophylaxis or Treatment^b^	ICH and GCS Deterioration	Treatment of ICH	Discharge Disposition and Postoperative Events	Follow-up duration (months)	Follow-up and GOS Changes
1	68 M	None	L VS; translabyrinthine	Prophylaxis: POD 1; enoxaparin	POD 2: IVH + SAH, hydrocephalus; ΔGCS score: 15 → worsened, unable to protect airway	EVD, VPS	Hospitalized 1 year: difficult VPS setting optimization features; GOS score: 4; SDH due to falls; left orbital fracture	93.6	GOS score: 3; disabled, bed-bound; dependent → died after 8 years due to complications
2	65 M	BMI 32	L VS; retrosigmoid; 344	Prophylaxis: POD 2; enoxaparin	POD 3: left ICH, 4.4 cm × 3.1 cm, mass effect; ΔGCS score: 15 → 3	EVD, VPS; multiple surgical evacuations	SNF → inpatient rehab → SNF; GOS score: 3; multiple episodes of altered mental status	21.3	ΔGOS score: 3 → 1; died within 2 years due to medical complications
3	43 M	BMI 30; smoker, 12.5 PY	L metastatic glomus tumor; retrosigmoid; 237	Prophylaxis: POD 1; enoxaparin	POD 3: left cerebellar hematoma, 4.6 cm × 2.7 cm; ΔGCS score: 15 → 9	Surgical evacuation; EVD	Home; GOS score: 5; motor weakness; L facial droop	6.5	GOS score: 5; returned to baseline
4	56 M	BMI 38	R VS; translabyrinthine; 585	Treatment: POD 5 UFH → enoxaparin + warfarin (POD 6) → IVC filter (postbleed)	POD 8: right CPA hemorrhage, 2 cm × 2 cm, mass effect; ΔGCS score: 15 → 9	Surgical evacuation; EVD	Inpatient rehab → home; GOS score: 4; recurrent VTEs: daily ASA, switched to rivaroxaban	15.1	GOS score: 5; returned to baseline, persistent dizziness
5	42 F	BMI 33; smoker, 4 PY; hyper–coagulopathy	R CPA meningioma; retrosigmoid; 239	Prophylaxis: POD 3 enoxaparin; VTE diagnosis POD 8/9; treatment: POD 9 UFH → warfarin/enoxaparin (POD 11)	POD 20: ICH; ΔGCS score: 15 → 3	Patient died in transit to the hospital	GOS score: 1	0.7	N/A

The ICH rate within this cohort was 2%. Notably, no mortality secondary to PE was identified in this cohort.

Case 1

**Figure 1:**
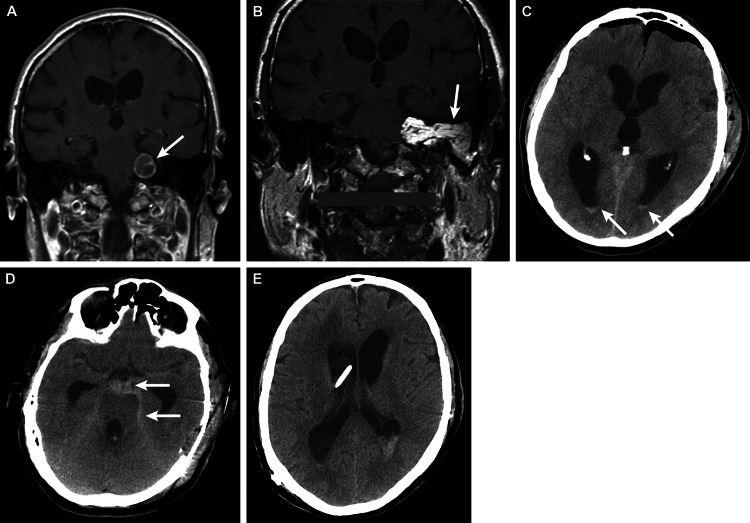
Case 1 – a 68-year-old man who presented with left-sided hearing loss, vertigo, and significant left-sided facial weakness (A) Preoperative coronal T1-weighted brain MRI displayed a left cerebellopontine (CP) angle-enhancing lesion (arrow), suggestive of a vestibular schwannoma, which measured approximately 1.7 × 1.7 cm, with no evident midbrain shift. (B) Postoperative coronal T1-weighted MRI showed expected postoperative changes from a left translabyrinthine craniotomy for resection. Fat packing could be visualized within the left CP angle and left mastoid air cells (arrow). (C and D) Axial CT on postoperative day two showed postoperative changes with no evidence of midline shift. (C) An intraventricular hemorrhage was revealed, and blood products could be seen in the occipital horns of lateral ventricles (white arrows), in addition to ventriculomegaly, indicating hydrocephalus. (D) A new perimesencephalic subarachnoid hemorrhage that extended to the suprasellar cisterns (arrows) could be detected. (E) Axial CT showing external ventricular drain placement in the frontal horn of the right lateral ventricle following intracerebral hemorrhage MRI: magnetic resonance imaging; CT: computed tomography Used with permission from Barrow Neurological Institute, Phoenix, Arizona

A 68-year-old man who presented with left-sided hearing loss, left-sided facial numbness, and balance difficulties was found to have a left VS measuring 1.7 × 1.7 cm (Figure 1A). Preoperatively, he had a House-Brackmann (HB) grade of I. He underwent an uncomplicated left translabyrinthine craniotomy for tumor resection. His immediate postoperative course went well, and he had a Glasgow Coma Scale (GCS) score of 15 and an HB grade of II (Figure 1B). Following standard protocol, DVT chemoprophylaxis with enoxaparin (40 mg, once daily, subcutaneous) was started 24 hours after the operation. Within the next 12 hours, the patient became lethargic and was found slow to follow commands with worsening facial droop. Imaging showed intraventricular and subarachnoid hemorrhage (Figures 1C, 1D), necessitating the urgent placement of an external ventricular drain, which was replaced with a ventriculoperitoneal shunt on postoperative day (POD) 23 (Figure 1E). The patient was discharged to a neurological rehabilitation center on POD 29, where he developed subdural hematoma due to falls, which required surgical evacuation. The subdural hematoma caused a decline in the patient’s neurological status, including difficulty in arousal, speaking, and swallowing.

The patient spent an additional six months as an inpatient secondary to shunt failure and failure to thrive. He faced significant difficulty in ventriculoperitoneal shunt setting optimization and continued to experience over-drainage and under-drainage of cerebrospinal fluid. Despite an endoscopic third ventriculostomy, the patient’s condition did not improve.

One year after the tumor resection, the patient developed a cerebrospinal fluid leak through the left ear and underwent defect repair with a dermal fat graft. Unfortunately, a left temporal venous hemorrhage on POD two worsened his already declining neurological status. Over the next seven years, the patient’s neurological status continued to decline, and he had multiple admissions for shunt repairs, hydrocephalus, seizures, and altered mental status. The patient lived in a group rehabilitation home and had a Glasgow Outcome Scale (GOS) score of 2 and a modified Rankin Scale (mRS) score of 5. He passed away after eight years.

Case 2

**Figure 2:**
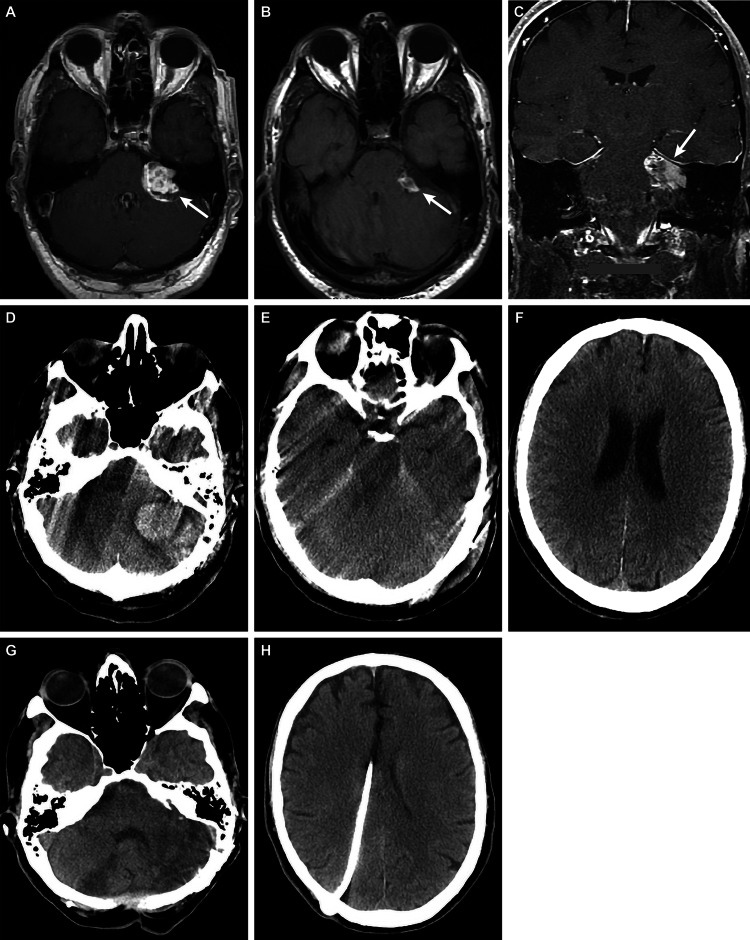
Case 2 – a 65-year-old man who presented with left-sided hearing loss (A) Preoperative axial T1-weighted MRI showed a 3.0 × 2.6-cm nonhomogeneous lesion in the cerebellopontine (CP) angle (arrow), suggestive of a vestibular schwannoma on the left labyrinth, compressing the brainstem. Postoperative (B) axial and (C) contrast-enhanced coronal T1-weighted MRI showed postoperative changes consistent with left retrosigmoid craniotomy for debulking of the left vestibular schwannoma. Fat packing could be seen (arrows). (D-F) Axial CT on postoperative day three showed postoperative changes and revealed (D) a new left posterior fossa hemorrhage measuring 4.4 × 3.1 cm, which caused cerebellar swelling, brainstem compression, fourth ventricle obstruction, (E) loss of basal cisterns, and (F) hydrocephalus. Axial CT, after multiple surgical interventions following a stroke, demonstrating (G) cerebellum resection and (H) ventriculoperitoneal shunt placement in the left lateral ventricle MRI: magnetic resonance imaging; CT: computed tomography Used with permission from Barrow Neurological Institute, Phoenix, Arizona

A 65-year-old man presented with left-sided hearing loss, and imaging revealed a large, 3.0 × 2.6-cm, left cystic VS (Figure 2A). The patient underwent a left retrosigmoid craniotomy for tumor resection. Immediate postoperative recovery was uneventful, and the patient had a postoperative GCS score of 15 and an HB grade of I (Figures 2B, 2C). In addition to receiving MP, the patient was started on subcutaneous enoxaparin (40 mg, once daily) in the evening of POD two (48 hours after the operation). However, the patient received a second dose in the late morning of POD three, only 18 hours after the first dose. Several hours later, the patient’s condition suddenly declined, and his GCS score was found to have decreased to 5 with bilateral loss of the corneal reflex. CT of the head showed a left posterior fossa hemorrhage with cerebellar swelling, brainstem compression, fourth ventricle obstruction, and hydrocephalus (Figures 2D, 2E, 2F). The patient’s hematoma was surgically evacuated and an external ventricular drain was placed. During the following week, the patient underwent six more procedures, including strokectomy and ventriculoperitoneal shunt placement (Figures 2G, 2H). On POD 18, after undergoing a tracheostomy and gastrostomy tube placement, the patient was transferred to a skilled nursing facility. However, he was soon readmitted to the inpatient rehabilitation facility because of his altered mental status. On POD 21 (post-bleed day 18), bilateral PEs and a left DVT were discovered, and treatment with UFH and an inferior vena cava (IVC) filter was commenced. However, a week later, heparin was discontinued because of gastrointestinal bleeding.

Later, the patient was discharged to a skilled nursing facility and physical therapy was started. During the next year, multiple hospital admissions were required due to the patient’s altered mental status. At the patient’s 22-month follow-up, he was found alert and oriented but was severely debilitated, with a GOS score of 3 and an mRS score of 5. His stroke sequelae included fixed aphasia, severe dysarthria, and aggressive behavior. He died in hospice care secondary to aspiration two years after the ICH.

Case 3

**Figure 3:**
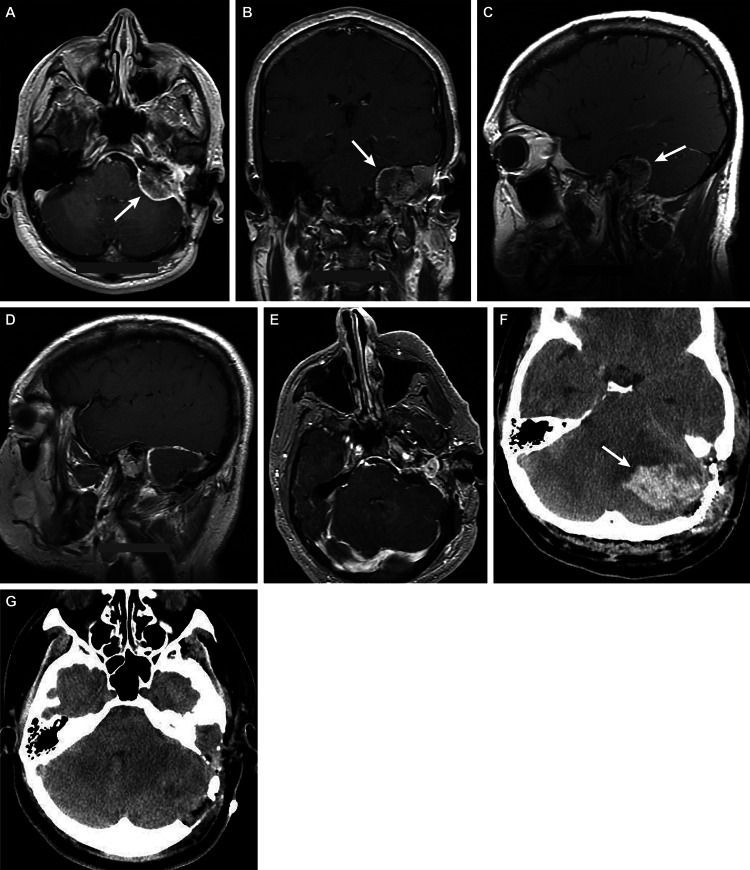
Case 3 – a 43-year-old man with a history of a recurring left metastatic glomus jugulare tumor (A) Axial CT and (B) coronal and (C) sagittal T1-weighted contrast-enhanced MRI indicated a left glomus jugulare tumor (arrows) measuring 3.3 × 4.7 × 4.1 cm in the anteroposterior, transverse, and craniocaudal dimensions. The tumor involved the petrous apex, with increased mass effect on the left cerebellopontine (CP) angle but no evidence of midline shift. The mass extended into the left middle ear, external auditory canal, and jugular foramen. Postoperative (D) sagittal and (E) axial T1-weighted contrast-enhanced MRI showed expected postoperative changes related to a left retrosigmoid craniotomy for debulking of the left CP angle mass. There was decreased mass effect, and a residual 1.6 × 1.7-cm nodular enhancement could be visualized in the resection cavity. (F and G) Axial CT on postoperative day three showed postoperative changes and revealed a new 4.6 × 2.7-cm left cerebellar hematoma (F, arrow), resulting in compression of the fourth ventricle and foramen magnum, brainstem (right midline) shift, and mild supratentorial herniation MRI: magnetic resonance imaging; CT: computed tomography Used with permission from Barrow Neurological Institute, Phoenix, Arizona

A 43-year-old man with a history of smoking presented with left-sided hearing loss, facial droop, and an HB grade of VI. He was a known case of metastatic glomus jugulare tumor and had undergone tumor resection and radiosurgery 11 years earlier. Surveillance scans showed a tumor measuring 3.3 × 4.7 × 4.1 cm extending into the middle and external ear, causing mass effect on the left cerebellopontine (CP) angle (Figures 3A, 3B, 3C).

A retrosigmoid craniotomy was performed to resect the tumor (Figures 3D, 3E). The patient recovered well, and DVT chemoprophylaxis with enoxaparin (40 mg, once daily) was initiated on POD one, approximately 31 hours after the operation. On POD three, 39 hours after the initiation of enoxaparin and after receiving two doses of the same, the patient became lethargic with slurred speech and developed a new left cranial nerve VI palsy, and his GCS score had decreased from 15 to 9. Diagnostic imaging showed a 4.6 × 2.7-cm left cerebellar hematoma that was causing the midline shift (Figures 3F, 3G).

The patient was intubated due to an acute decrease in respiratory function and underwent hematoma evacuation. The patient recovered uneventfully and was discharged from the hospital on POD 17. At the three-month clinical follow-up, the patient reported behavioral issues (blurting out words, anger, and impulsivity), which was found to be improved at the six-month follow-up, and his neurological status returned to the baseline, with an mRS score of 1 (GOS score of 5).

Case 4

**Figure 4:**
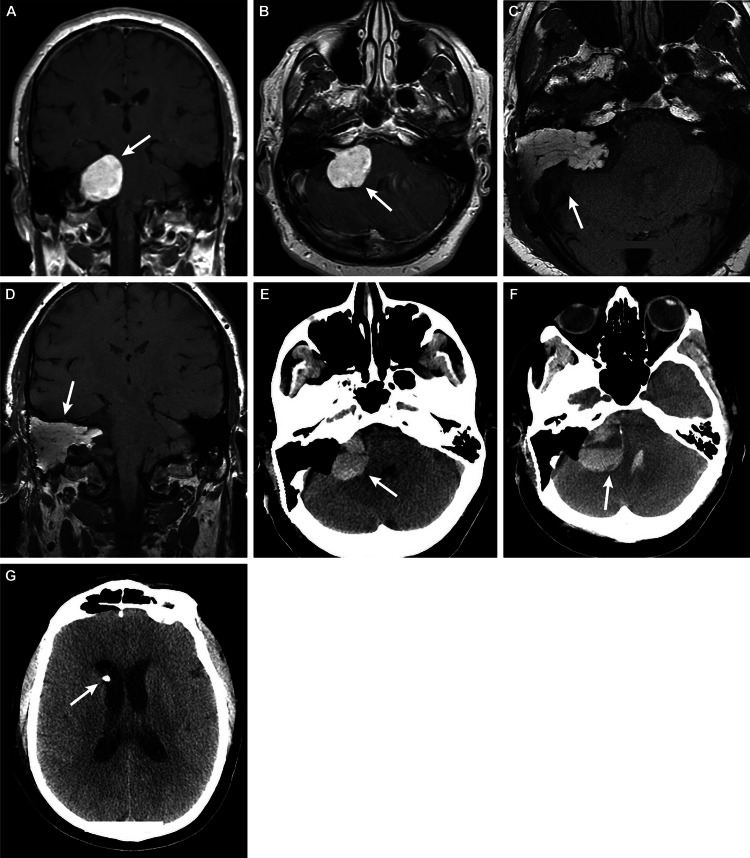
Case 4 – a 56-year-old man who presented with right-sided hearing loss Preoperative (A) coronal and (B) axial T1-weighted MRI indicated a right vestibular schwannoma (arrows) measuring 3.7 × 3.8 × 3.3 cm, causing brainstem compression and mass effect on the right cerebellum. Postoperative (C) axial and (D) coronal T1-weighted MRI demonstrated expected postoperative changes related to a right translabyrinthine craniotomy for resection of the large vestibular schwannoma, with fat-packing (arrows) in the mastoid air cells. Mass effect on the right brainstem and cerebellum was not significantly changed. (E) Axial CT on postoperative day nine showed postoperative changes and a new 3.4 × 2-cm hyperdensity (arrow) consistent with a hemorrhage in the right cerebellopontine (CP) angle operative bed. It indicated an increased mass effect on the right middle cerebellar peduncle and pons and on the fourth ventricle. (F) Axial CT performed on postoperative day 10 showed evidence of an expanding hematoma (arrow), now 4 × 3 cm, causing further increased mass effect. Blood products could also be viewed in the fourth ventricle. (G) Axial CT showed the placement of an external ventricular drain (arrow) in the frontal horn of the right lateral ventricle following the surgical evacuation of the hematoma MRI: magnetic resonance imaging; CT: computed tomography Used with permission from Barrow Neurological Institute, Phoenix, Arizona

A 56-year-old man with a BMI of 38 kg/m^2^ presented with unilateral hearing loss. Imaging revealed a large right VS measuring 3.7 × 3.8 × 3.3 cm causing brainstem compression (Figures 4A, 4B). 

The tumor was resected via a translabyrinthine approach (Figures 4C, 4D). The patient’s immediate postoperative GCS score was 15, and his HB grade was II. On POD three, the patient reported bilateral leg pain and swelling. Doppler ultrasound showed bilateral lower extremity DVT. Anticoagulation was not administered because of a recent cranial procedure. On POD five, the patient was started on a prophylactic dose of UFH (5,000 U, subcutaneous, twice daily) after reporting mild chest pain, although the patient was ambulating. Later at night, the patient experienced severe chest pain, and CT of the chest revealed bilateral PE. The patient’s regimen was immediately switched to a therapeutic dosage of enoxaparin (130 mg, twice daily; recommended dose: 1 mg/kg) while therapy was bridged to warfarin (7.5 mg, once daily).

On POD eight, after receiving the fifth dose of enoxaparin, the patient developed a severe headache and nausea with bleeding from the operative site. An immediate CT of the head showed no evidence of ICH. However, the patient’s headache worsened, and a repeat CT of the head demonstrated a right CP angle hemorrhage that was 3.4 × 2 cm in size, causing compression of the cerebellum, pons, and fourth ventricle (Figures 4E, 4F). The patient received an IVC filter and protamine for heparin reversal after the discontinuation of anticoagulants. The patient remained neurologically stable and was closely monitored. On POD 10, the patient’s GCS score was found to have decreased to 9, and CT of the head revealed the increasing size of hematoma (now 4 × 3 cm) with mass effect, intraventricular hemorrhage, and hydrocephalus, necessitating surgical evacuation and placement of an external ventricular drain (Figure 4G).

Two weeks after the ICH, the patient was discharged to the neurorehabilitation facility, and his condition improved with physiotherapy. Doppler ultrasound was performed regularly for DVT surveillance. Three months later, rivaroxaban was initiated after venous sinus thrombosis was discovered on imaging. At the one-year follow-up, the patient was found to be well and had returned to neurological baseline (mRS score of 1; GOS score of 5).

Case 5

**Figure 5:**
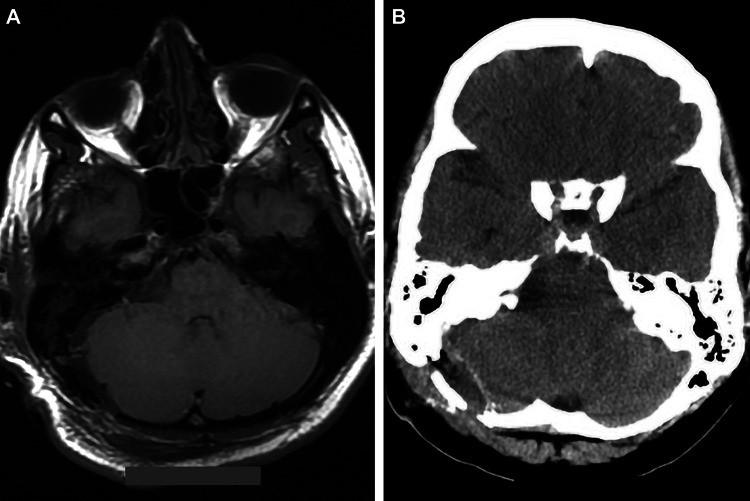
Case 5 – a 42-year-old woman with a known case of hypercoagulopathy who presented with right-sided hearing loss and facial weakness and received a diagnosis of right cerebellopontine (CP) angle mass (A) Postoperative axial T1-weighted MRI showed changes consistent with a retrosigmoid craniotomy for resection of a right cerebellopontine (CP) angle mass. (B) Axial CT from postoperative day nine, performed because of a syncopal episode, demonstrated stable postoperative changes and a stable amount of extra-axial fluid and gas deep in the craniotomy site. There was no evidence of a superimposed acute intracranial process MRI: magnetic resonance imaging; CT: computed tomography Used with permission from Barrow Neurological Institute, Phoenix, Arizona

A 42-year-old woman with a known case of hypercoagulable disorder and a history of smoking presented with dizziness, blurred vision, headaches, right-sided hearing loss, and facial weakness (HB grade II). She had experienced multiple episodes of DVT, PE (IVC filter in place), and myocardial infarction at a young age, for which she was taking warfarin with an international normalized ratio goal of 3. She received a diagnosis of a right CP angle mass measuring 2.4 × 2.6 × 2.8 cm (imaging unavailable) and underwent retrosigmoid craniotomy for tumor resection (Figure 5A). Histopathology findings were consistent with a meningothelial meningioma. The patient recovered well and subsequently had an HB grade of I. On POD three, because of her medical history, the DVT prophylaxis with enoxaparin was initiated. On POD nine, she experienced sudden tachypnea, tachycardia, and syncope. CT angiography of the chest showed bilateral PEs, and findings from CT of the head did not indicate ICH (Figure 5B).

Doppler ultrasound demonstrated left popliteal vein nonocclusive thrombosis. Intravenous heparin drip was initiated (target range of partial thromboplastin time: 40-60 seconds) without loading bolus due to the recent cranial procedure. On POD 11, repeat CT angiography showed mild right heart strain, which prompted the anticoagulation treatment plan to be switched from heparin to enoxaparin (90 mg, twice daily) while being bridged to warfarin (7.5 mg, once daily). On POD 12, the patient was discharged home and was educated about international normalized ratio monitoring. On POD 20, the patient presented to an outside hospital with a small ICH (imaging unavailable). Unfortunately, the hematoma grew, and the patient’s GCS score decreased to 3 during her transfer to a tertiary care center, and she was pronounced dead on arrival (mRS score of 6; GOS score of 1).

## Discussion

We described five cases of posterior fossa hemorrhage after the use of a therapeutic or prophylactic dose of anticoagulants. Four of the five patients had debilitating outcomes, with three having GOS scores of ≤3. All four of these patients had immediate postoperative GCS scores of 15 and were recovering well from their surgical procedures. All four patients with debilitating outcomes received prophylactic doses of LMWH within three days of surgery. Interestingly, Carlson et al. [24] identified anticoagulation as a strong predictor of intratumoral hemorrhage in patients with VS (without a history of craniotomy), and they reported that the administering of anticoagulation drugs was associated with a 25-fold increase in risk. These findings hint at the unique cytoarchitecture and microanatomy of the posterior fossa.

**Figure 6:**
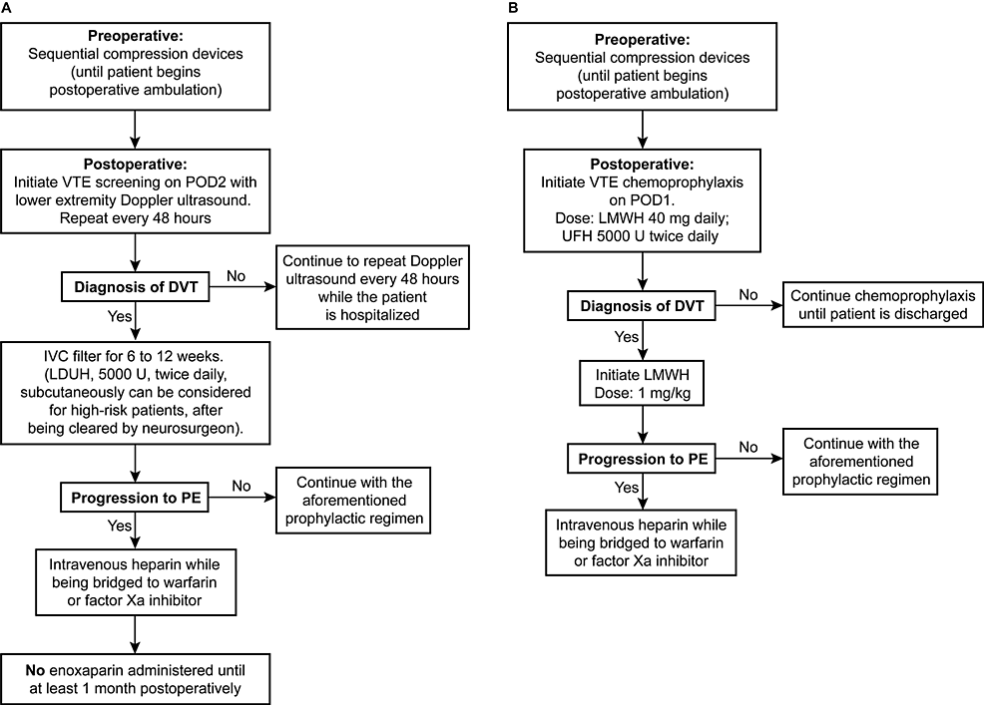
Algorithms (A) Algorithm for the current prophylaxis and treatment of VTE following posterior fossa craniotomy. (B) An algorithm outlining the previous practice of VTE prophylaxis VTE: venous thromboembolism; DVT: deep venous thrombosis; IVC: inferior vena cava; LDUH: low-dose unfractionated heparin; LMWH: low-molecular-weight heparin; MP: mechanical prophylaxis; PE: pulmonary embolism; POD: postoperative day; UFH: unfractionated heparin Used with permission from Barrow Neurological Institute, Phoenix, Arizona

In this case series, we managed VTE according to the established standard of care, which resulted in debilitating outcomes for our patients. All patients had postoperative GCS scores of 15 and good facial outcomes, indicating the likelihood of a favorable outcome. However, for three of the five patients, this status changed rapidly to chronic disability or death. These outcomes led us to critically analyze the existing recommendations for VTE management and to search for evidence from the existing literature. We discovered that the level of available evidence was grade 2C. Moreover, the existing literature does not differentiate between posterior fossa lesions and supratentorial lesions, which raises concerns regarding generalizability. Therefore, we revised our postoperative care practices for VTE prophylaxis and treatment (Figure 6A).

The goal following any craniotomy should be to mobilize the patient as soon as possible, in addition to providing MP and chemoprophylaxis with the caveats detailed below.

Lessons learned

We recommend that all patients should undergo leg compression via sequential compression devices and/or compression hose in the preoperative holding area on the day of surgery, which should be continued intraoperatively and postoperatively until the patient starts ambulating at least three times per day. In the operating rooms, care should be taken when positioning the patient to maximize venous return and prevent stasis, which is a key promoter of coagulation. For patients who undergo posterior fossa surgery, we propose the cautious use of anticoagulation to prevent intracranial bleeding as described in the following section.

Posterior fossa craniotomy

We advocate postoperative DVT screening with duplex ultrasound of the lower extremities performed every 48 hours until the patient starts ambulating. In the case of a DVT diagnosis, we recommend the placement of an IVC filter. For patients at high risk, UFH can be administered if there is consensus among the multidisciplinary team. We recommend a dosage of 5,000 U twice daily instead of three times daily because the literature does not report any significant difference in the efficacy between these two regimens [25]. Conversely, in the case of PE, treatment with heparin drip and warfarin or factor Xa inhibitor should be initiated. We do not recommend LMWH in any form or at any dosage (prophylactic or therapeutic) until at least one month after surgery (Figure 6A). For the purpose of comparison, an algorithm outlining our previous practice shows early postoperative use of LMWH (Figure 6B).

LMWH is associated with an increased risk of death from any cause when used for DVT prophylaxis [21]. It is estimated that LMWH prevents between 8-36 cases of VTE per 1,000 patients at the expense of 4-22 additional cases of ICH per 1,000 patients [21]. On the assumption that the disutility associated with ICH is two to three times greater than that associated with VTE, the use of LMWH causes more harm than benefit [21]. The Joint Commission on Accreditation of Healthcare Organizations monitors hospital VTE rates as a quality-control measure [26], and this serves as a major driving force behind efforts to reduce VTE rates. However, the prevention of VTE may occur at the cost of an increase in the cases of ICH, which is generally more debilitating than VTE and more likely to be fatal. Therefore, before initiating LMWH among patients after a posterior fossa craniotomy, it is critical to consider whether the prophylactic treatment entails a risk of a debilitating ICH while preventing only a thromboembolic event with no major sequelae.

Use of mechanical prophylaxis and chemoprophylaxis

A landmark randomized controlled trial (RCT), published in 1998, compared the efficacy and safety of compression stockings alone with that of enoxaparin plus compression stockings for VTE prevention in elective neurosurgery [12]. The trial included 307 patients and reported that enoxaparin plus compression stockings not only prevents VTE but also does not increase the risk of ICH. This result was supported by several studies that proved the efficacy of enoxaparin [13,27,28]. The highest level of evidence available for this finding is class 2C, which is a weak recommendation favoring clinical judgment [21]. Danish et al. [22] also raised an interesting point with regard to the feasibility of conducting an RCT with three treatment arms (i.e., LMWH + MP, UFH + MP, and MP alone) to produce class I evidence. The sample size that would be required with current indices of DVT, PE, and ICH is 38,000 cases. Because such a large RCT is impractical, clinical judgment must play the primary role in deciding between the different modalities. A recent systematic review by Algattas et al. [4], published in 2018, analyzed all previous studies and compared the efficacy, safety, and cost-effectiveness of LMWH + MP, UFH + MP, and MP alone for the prevention of VTE among patients undergoing craniotomy for a brain tumor. In total, 34 studies were reviewed. Algattas et al. [4] reported the risk of VTE as 2.78% (95% CI: 1.23-5.15%) among patients with LMWH + MP, as 1.49% (95% CI: 0.42-3.72%) among patients with UFH + MP, and as 2.59% (95% CI: 1.31-4.58%) among patients with MP alone; the risk of ICH among these groups was reported as 2.72% (95% CI: 1.23-5.15%), 0.74% (95% CI: 0.09-2.61%), and 0.26% (95% CI: 0.01-1.34%), respectively. However, the authors found no statistically significant difference between the rates [4]. They concluded that UFH + MP is the safest and most cost-effective method for VTE prevention following cranial surgery [4]. In our study, in all three cases involving debilitating outcomes/death, LMWH was initiated in the early postoperative period. Based on the data in the literature and our own experience, we conclude that the use of UFH is preferred.

Timing of chemoprophylaxis

The timing of anticoagulation initiation has been identified as a predictor of ICH and needs to be considered while weighing the benefits associated with early initiation against the risks. Published data suggest that the majority of ICH cases occur within 12-24 hours following surgery, whereas most VTE cases develop one week after surgery [3,21]. Therefore, we recommend a delay in the use of chemoprophylaxis. We report a higher incidence of ICH resulting in debilitating outcomes with the initiation of enoxaparin in the early postoperative period. Similar findings were reported in an RCT conducted to study the role of enoxaparin versus sequential compression devices (SCDs) for DVT prophylaxis when given preoperatively in 68 patients undergoing surgery for intracranial neoplasm [16]. The incidence rate of DVT remained the same in both groups. However, the rate of postoperative ICH was reported to be as high as 11% in the enoxaparin group, whereas no cases of ICH were reported in the sequential compression device group. The study was terminated because of a high rate of adverse outcomes [16]. Another study compared postoperative bleeding among patients who underwent craniotomy and received perioperative prophylactic doses of UFH (55 patients) versus placebo (48 patients). This study used postoperative red blood cell mass loss as an indicator of bleeding and did not report any significant difference between the treatment arms [29].

Further studies are required to calculate ICH and VTE rates with and without prophylactic anticoagulation after posterior fossa craniotomy. It is critical to analyze VTE and ICH outcomes and implement the conclusion drawn through prospective studies.

Part of the content of this paper was presented at the North American Skull Base Society 29th Annual Meeting, February 15-17, 2019, Orlando, Florida.

## Conclusions

We reported five cases of ICH following the use of anticoagulation for VTE treatment and prophylaxis. This study highlights debilitating outcomes, including death, associated with the use of anticoagulation drugs in the early postoperative period. LMWH is associated with an increased risk of ICH following surgery, especially in cases of posterior fossa pathology. Therefore, we recommend the use of UFH rather than LMWH in the postoperative period and initiating the use of SCDs preoperatively, which should be continued until the patient starts ambulating. Further studies are required to more comprehensively assess the outcomes related to VTE and ICH with the use of LMWH and UFH in posterior fossa surgical procedures. Clear guidelines need to be established for the use of anticoagulants among patients who undergo posterior fossa craniotomy.
